# From Value to Valuation: Pragmatist and Hermeneutic Orientations for Assessing Science on the International Space Station

**DOI:** 10.1007/s12108-021-09515-y

**Published:** 2021-10-30

**Authors:** Paola Castaño

**Affiliations:** School of Social Sciences, Cardiff University, Glamorgan Building, King Edward VII Avenue, Cardiff, CF10 3WT United Kingdom

**Keywords:** Valuation, Hermeneutics, Pragmatism, Sociology, Science, Institutions, International Space Station

## Abstract

Based on a study of the International Space Station (ISS), this paper argues that – as a set of orientations for sociological inquiry – pragmatism and hermeneutics are confluent frameworks to examine valuation as a social process. This confluence is grounded on their common attunement to valuing as a problematic and relational process, their equally common updates with theories of institutions, and a further conceptual development regarding the temporalities of valuation. I advance the argument in four steps. First, looking at how the question about the “scientific value” of the ISS is far from settled, I show how valuation is always about something considered problematic and indeterminate. Second, characterizing the ISS at the intersection of different criteria of assessment, I stress the nature of valuation as a fundamentally perspectival and interpretive process, and show how a hermeneutic approach can complement some of the limitations of pragmatism in this regard. Third, I look at the question of institutions considering how some modes of assessment sediment more successfully than others. Fourth, I argue that, while providing insights towards it, pragmatist and hermeneutic approaches to valuation have not fully grasped its temporal nature as a process, and outline ways to open this line of inquiry. I conclude with some ideas for studies in sociology of science to re-entangle detailed case studies of scientific practice with the study of how institutions make claims of worth about the nature of science, I propose ways to extend these arguments to other studies of what I call *iridescent institutions*, and I make some considerations about our stance as sociologists in these valuation disputes.

“Ladies and gentlemen, we have detected gravitational waves! We did it!” With those words, the executive director and scientific spokesman of the Laser Interferometer Gravitational-Wave Observatory (LIGO), cheerfully announced the first detection of gravitational waves at a press conference hosted by the National Science Foundation in Washington, D.C. on February 11, 2016. A few years before, in 2012, the director-general of the European Organization for Nuclear Research (CERN) said “We have it!” in the midst of cheers as he announced the discovery of the Higgs boson. More recently, in April of 2019 at seven simultaneous press conferences, members of the Event Horizon Telescope’s collaboration unveiled the first ever image of a black hole. As media reports followed with various details about the process that led to the discoveries (Castelvecchi & Witze, 2016; Biever, 2012; Butterworth, 2015; Banks, 2019), these moments marked the indisputable culmination of decades of work in these large collaborations enabled by equally large infrastructures. And, in more prosaic terms, had there been any dissenting voices about how “worth-the-money” these large projects were, the announcements above quite possibly quieted them.

The International Space Station (ISS), conceived as “a world renowned laboratory in space enabling discoveries in science and technology that benefit life on Earth and exploration of the universe” (NASA, 2015), has not had one of those “ta-da!” moments. What follows does not interpret that fact in terms of a failure and, like everything related to the ISS, offers a “it is more complicated” story. The complications result from questioning the very grounds of comparison between the ISS and “big science,” from acknowledging that – in spite of that fact – the station cannot easily escape those comparative framings and their expectations, and from shifting focus from value to valuation as a social process.

Based on a study of the National Aeronautics and Space Administration’s (NASA) portion of the ISS (Castaño, forthcoming), this paper argues that – as a set of orientations for sociological inquiry – pragmatism and hermeneutics are confluent frameworks to examine valuation as a social process. This confluence is grounded on their common attunement to valuing as a problematic and relational process, their equally common updates with theories of institutions, and a further conceptual development they require about the temporalities of valuation.

The ISS is the most expensive, complex, and sustained effort to put humans in space and responds to heterogeneous criteria of value assessment. In the agreements signed in 1998 by the U.S, Russian, European, Canadian, and Japanese space agencies, the creation of “a civil international Space Station for peaceful purposes” (Agreement, 1998) was justified in terms of various goals and, in consequence, the station involves an array of functions and promises. It is an engineering feat as the largest human-made object outside this planet (orbiting Earth at an altitude of 250 miles and a speed of 17,100 miles per hour) with a total pressurized volume of 931.57 m^3^. It is also the most wide-ranging international cooperative space program in history; a test bed to develop technologies both for “Earth” and space exploration to the Moon and Mars; a proving ground for private companies to create a commercial market in Low Earth Orbit (LEO) as governmental funding is set to cease in 2024; an outpost with inspirational and educational roles; and an orbiting laboratory (Castaño, 2020). Since it became fully operational in 2011, it has enabled 2,179 experiments (NASA, 2021) in multiple fields, but the question of whether it has fulfilled its scientific ambitions remains contentious.

“What is the scientific value of the International Space Station?” That is a pressing and consequential concern given the station’s total estimated cost of $150 billion and annual operations costs of approximately $3 billion for the United States alone. The question is inescapably embedded in two larger issues: “Is the ISS really worth it?” and “Is there a compelling rationale for human spaceflight?” Posed in those terms, and in relation to those two much thicker questions, the puzzle of the scientific value of the ISS presupposes a kind of transparency in its formulation as separated from standpoint. And there is nothing but standpoints as it comes to these issues. The absence of a “ta-da!” moment makes it all particularly complex and, precisely because of that, makes it a sociologically fruitful research site to consider the puzzles of valuation.

For NASA, the urgency about the formulation of scientific value for the ISS is clear since the stakes are budgetary allocations and the very the future of the program and of research in human spaceflight more broadly: “Longevity of research programs is driven by results because results drive policies that strategically fund the most promising research. Therefore, it is critical to communicate the breakthroughs and benefits that result from ISS research in ways that are relevant to all ISS stakeholders” (Ruttley et al., 2017, 1163). The issue, however, is that the ISS is held accountable by decision-makers, participants and audiences with diverse criteria and expectations which makes its valuation highly contentious.

In the most general terms, the station is surrounded by supporters and critics that – with varying types of involvement, expectations, and entitlements – selectively highlight different aspects of the broad portfolio of activities and experiments on the station to make their case. In ideal-typical terms, the first (which clearly include the spokespeople for the program) think that human spaceflight is a vital activity in light of ideas of exploration, discovery, and inspiration; and the second place the ISS’ price tag next to “proper big science” (that of CERN, LIGO, the Hubble Space Telescope, and other space probes) in whose light, via the usual metrics of operations costs, number of publications, and major discoveries, the station looks disappointing.

Space historians and space policy experts have dwelled on the issue of public support for space activities for years (McCurdy, 1990; Logsdon, 2005; Launius, 2006, 2017), and they frequently do so naturalizing some of NASA’s own categories. That is, invoking a notion of “the American public,” arguing for or against a set of rationales for human spaceflight, taking for granted that “return on investment” is the unavoidable framework of assessment, and ultimately relying on tactical – almost “realpolitik” – assumptions about the actors and institutions involved.

My attempt here, and in my overall approach to the ISS, is to offer a sociologically detailed approach to these issues following specific processes and actors in their diverse modes of understanding. Investigation into practices of assessment must rely on a substantive understanding of that which is being assessed, and that is why my ethnographic, archival, and interview-based examination of experiments over six years is the groundwork for this paper (Castaño, forthcoming). From there, it is possible to engage in a rigorous inquiry of valuation considering who is doing the assessment work, from which institutional stance, based on what kind of understanding of science, using which types of evidence, and with what kind of vision about the station’s endeavor.

The growing interest in the sociology on valuation (Helgesson & Muniesa, 2013; Kjellberg & Mallard, 2013; Antal et al., 2015; Heinich, 2020a, b) does not represent a unified field. Lamont identified eight literatures in sociology that address valuation, but also a common ground among them: they are “concerned with how value is produced, diffused, assessed, and institutionalized across a range of settings” (2012, 203). Another common ground is that these studies acknowledge their intellectual roots in pragmatism, specifically in the work of John Dewey (1939). However, they do not place themselves explicitly in relation to hermeneutics as a broader intellectual tradition despite having clear affinities with it.

The labels pragmatism and hermeneutics certainly accommodate different strands of thought (Ogien, 2015, 2) which are also far from homogeneous and have had diverse uptakes in the social sciences (Oliver, 1983; Alexander, 1987; Ogien, 2014; Babich, 2017). In what follows, my takes on pragmatism and hermeneutics are specific in their genealogy and wide-ranging in their implications. I situate my use of pragmatism in a conceptually principled and methodologically open-ended interpretation of Dewey’s theory of valuation (1939) whose main move entails turning the attention away from value or values and towards valuation as a process. As I describe in what follows, different sociological agendas about valuation, as well as questions about the fulfillment of the pragmatist ambition (Muniesa, 2011), have emerged and can emerge from this key starting point.

Regarding hermeneutics, I focus on the Diltheyan thread of what Oliver (1983) calls “old hermeneutics” which also includes Giambattista Vico and Johann Gottfried Herder. In its most general formulation, Dilthey situated understanding and interpretation as the foundational epistemological problems of the *Geisteswissenschaften* (1900, 252-253). Considering discrepancies about the degree of empathic and experiential involvement in that understanding and interpretation (as well as in their explanatory nature), this line of thinking finds a sociological resolution in Max Weber’s view of sociology as an empirical discipline based on *verstehen,* and – from there – in the broad camp of interpretive sociology (Martin, 2000). This line of engagement deliberately leaves aside the “new hermeneutics” defined by Oliver as a twentieth century product and a strand that follows Heidegger’s philosophy.

With these demarcations in mind, I situate pragmatism and hermeneutics as theoretical orientations that inform my overall approach to the ISS, to valuation, and to sociological inquiry in general. First, they have processual ontologies; second, they are untroubled by the diversity and complexity of social life and do not treat them as something to be overcome or “resolved;” third, they acknowledge that all social actors are constantly engaged in interpretive activities that involve considerations of good/bad, better/worse and right/wrong; and fourth, they take on interpretive activities empirically via systematic interpretation.

From these premises, I characterize valuation as a process that is problematic, indeterminate, relational, and perspectival, and that needs to incorporate an account of institutions and time. Starting with Dewey’s move, value is not a property, but it is always connected with an activity, a process of apprehension (1939, 4). Clearly, from a hermeneutic standpoint that is of assistance here, these operations are never unanimous, do not result in a once and for all settled issue, and the very aspects of the object under valuation cannot be taken for granted. One crucial question, therefore, is about how valuing practices are institutionalized and to which institutions they give rise. Both hermeneutics and pragmatism have precisely been revisited in sociology the last decade with perspectives about institutions with the works of Glaeser (2011, 2014) and Bargheer (2018) respectively. They represent a revival of these two traditions in our discipline crucially expanding the grounds of conversation about institutions.

Building on these two updates, I show how valuing as a fundamentally institutional process is also a time-oriented endeavor in which what ought to be is always outside the present. I develop this argument in four steps that entail a characterization of the puzzles set by the ISS in relation to central pragmatist and hermeneutical arguments relevant to valuation. First, looking at how the question about the “scientific value” of the ISS is far from settled, I show how valuation is always an inquiry about something considered problematic and indeterminate, and precisely there lies its importance. Second, characterizing the ISS at the intersection of various frameworks of assessment, I stress the nature of valuation as a fundamentally perspectival and interpretive process. I exemplify this point with a hearing at the Committee on Science, Space and Technology in the U.S. House of Representatives, a setting where the contentiousness of valuation is ritualized and becomes pointedly clear. Third, I look at the question of institutions considering how some modes of assessment – in this case, those based on “return on investment” – sediment more successfully than others over time. Fourth, I argue that, while providing insights towards it, pragmatist and hermeneutic approaches to valuation have not fully grasped its temporal nature as a process. I conclude with some ideas for studies in sociology of science to re-entangle their detailed case studies of scientific practice with the study of how institutions make claims of worth about the nature of science, I propose ways to extend these arguments to other studies of multi-jurisdictional institutions, and I make some considerations about our own stance as sociologists in these valuation disputes.

## The International Space Station

The question about the “scientific value” of the ISS is contentious and far from settled. As most matters of contention, a significant part of the issue lies on who enters in the discussion and what they are actually considering. The complexity of the space station cannot be overstated, and all assessments of its science inexorably involve assessments of its overall endeavour. With these premises in mind, there are four major characteristics of the ISS relevant to the issue of valuation.

### Promises and Pressures

The ISS was initially envisioned in the 1980s as NASA’s *Space Station Freedom*, and as a straightforward Cold War instrument of “U.S. strength in space.” From there, at least seven major redesigns followed until the program survived by merely one vote in Congress in 1993, when the rhetoric of leadership gave way to one of international cooperation, not only with the partners that had already been invited to participate (Europe, Japan, and Canada), but also with a former political enemy (Russia).

The ISS is the result of a political agreement signed in 1998 and followed by multiple implementational arrangements, logistical alliances, and budgetary negotiations (Logsdon, 1998; Moenter, 1999; Sharpe & Tronchetti, 2015). In each of these steps, the station was justified before different decision-makers, scientists, participants, and audiences as a means for the fulfillment of their various goals and its potential was consistently overstated. In Neal’s words, “space historians and policy analysts have expressed wonder that this space station survived its long turmoil” as NASA “had to promise to do more with less in a risky spiral of overselling” (2017, 13).

The hyperbolic framings of the station as universally beneficial – the “pyrotechnics of promise” as I call them – and the multiplication of ends, means, and stakeholders necessitated by the sheer magnitude of the project are the crucial starting points to understand the ISS. However, as bold as the imagined futures of exploration seemed in the 1980s, there are various sobering facts described by a NASA Office of the Inspector General (OIG) report: “Final configuration of the ISS cost more, took longer to complete, and is less capable than planners envisioned” (2013, 2). When the station was initially proposed, the estimations were for complete assembly by 1994 at a cost of $8 billion. In the end, construction of the ISS was completed in 2011 with a cost of $100 billion. In addition, while having seven functions next to that of the laboratory, the length of the ISS decreased from 493 to 357 feet (a 27.6% reduction compared to earlier plans). The OIG report concluded that “cost growth, schedule delays, and functional changes resulted primarily from underestimating the technical complexity of building the Station; changes in Federal government funding priorities; and the Challenger and Columbia Space Shuttle accidents which delayed and reduced the number of Shuttle flights devoted to ISS assembly” (Ibid, 9).

As a result of its tumultuous process of approval and assemblage, the program was under great pressure from the start and, even before completion of assembly, questions about its scientific results were already on the table. A 2005 NASA Authorization Act designated the U.S. segment of the ISS as a National Laboratory, and five years later, a new organization – CASIS (Center for the Advancement of Science in Space) – was designated to manage it. In practice, the process has entailed a split of 50% of all U.S resources on the ISS between NASA (with a focus on exploration-oriented research) and CASIS (with a focus on research that benefits Earth and involvement of private actors and non-traditional users of space with the station). Given the newness of this model and the import of its mandate, the entire process has been labyrinthine, and a recent report by an Independent Review Team (2020) has suggested changes to their working model.

### Routine and Process

Unlike a Moon landing, a space station does not provide a single flag-planting moment that can be easily photographed. Although there have been milestones both in terms of assemblage and operations, the overall endeavor is not about major events but about multiple and complex processes, and routinization is, in fact, its main accomplishment. The ISS is part of an era in which spaceflight has become routinized and, as Lewis puts it, “most missions lack the drama of earlier space flight that captured public interest and political support” (2014, 22).

When it comes to its scientific activities, the same holds true. Given existing criteria of newsworthiness in science reporting (Harcup & O’Neill, 2001; Franklin, 2002; Neil et al., 2005), not much from the ISS lends itself to an easy headline. There is a particular thirst for the idea of breakthrough as an event and, moreover, as a spectacle frequently leading to amplifications or misrepresentations of the nuances of findings on the station (Brumfield, 2013; Koren, 2018). In consequence, by placing the station’s justification entirely in the realm of large ends, the process and various ends-in-view (using Dewey’s term) of the program have been placed in the almost invisible background for science valuation processes.

### Multipurposefulness and the Role of Science

The ISS is a multi-purpose program and facility where science is just one of its activities. This is not merely the context or background against which scientific research occurs, but it fundamentally shapes what scientific research is and can be on the station. While “science” added a unique weight to the moral claim of necessity for a space station, the ISS as a laboratory would not exist in the absence of other promises across domains (international cooperation, commercial profit, public support, educational inspiration, and technological development). And in the context of operations, science is - again - only one operation amongst others.

Historically, the dichotomy between field and laboratory has served to demarcate the sites of “actual” scientific practice as distinct from those of “mere” data collection (Heggie, 2014). Studies of extreme environments have problematized this demarcation showing that in polar and tropical field stations the laboratory and field are the same, and that the separation between habitation and work is also diluted. Geissler and Kelly (2016) have shown how these stations, as sites for visiting scientists, have to combine the routines of everyday life with those of research. In the case of Antarctica, Salazar has illustrated how this environment brings to the fore the “relational conditions” of life that would otherwise be more compartmentalized (2017, 152). In sum, these locations blur “almost all categorical assumptions: field, site, laboratory, instrument, nature and artifice are all blended into a single home for the production of scientific knowledge” (Heggie, 2016, 826).

The ISS shares all those features with these Earth-based field stations, but two critical and unique features further complicate the picture. The first is the entirely self-enclosed and artificial nature of “the field” and the essential role of microgravity in shaping all aspects of everyday life on the station. On the station, astronauts not only need to go through intense physical and psychological adjustments to confinement and microgravity, but also quotidian objects and tasks related to experiments and life in general must be made suitable for this environment via explicitation and instruction (Liverman et al., 2012; Castaño, forthcoming). The second major difference between the ISS and other research stations in extreme environments is that, in space, “the expeditioners” – usually six crew members at a given time – are scientific technicians by delegation who perform a variety of operational tasks.

There is a significant consequence for the problem of assessment here that, more than a critical punchline, should be the starting point of any assessment of scientific experimentation on the ISS: the station was not built only for science, and it is not a “big science” project. For this characterization one cannot simply look at costs and infrastructures, but at the entire process of conducting experiments on the ISS. Three features summarize this process. First, the diversity of fields of research covering experiments based on automated data collection using the platform (the only ones that could resemble “big science”), experiments involving astronauts acting as laboratory technicians collecting data, and experiments involving crew members as test subjects. Second, the time and logistics by which experiments make it to the ISS are diverse and, in most cases, cumbersome (Kiss, 2015). And third, the nature of activities in which crew members are involved revolve around guided data collection and  multiple housekeeping tasks that make their on-board life possible. Bluntly put, in most big science projects or National Laboratories, the individuals in charge of data collection are not the same ones in charge of fixing the toilet in their facilities on a regular basis, handling the trash, nor flying the vehicles that take them to the location.

### Diverse Stakeholders with Equally Diverse Criteria to Assess Worth

Given its broad promises and areas of operation, the ISS is embedded in what, using Boltanski and Thévenot's (2006) terminology, is a plurality of grammars of evaluation. Regarding research, the station holds the heavy weight of accountability for its “science return,” and articulates it in terms of benefits and impact. Program officials characterize the complexity of the undertaking in these terms: “Because of the unique microgravity environment of the ISS laboratory, the multidisciplinary and international nature of the research, and the significance of the investment in its development, analyzing ISS scientific impacts is an exceptional challenge” (ISS Program Science Forum, 2018, 39). More specifically, the task entails, again in their words, developing “appropriate metrics to communicate the impacts of ISS utilization, which have come in both tangible and intangible forms” (Ruttley et al., 2017, 1163).

The program uses two main instruments for this communication: the book *ISS Benefits for Humanity*, which is now in its third edition, and the *Annual Highlights of Results from the ISS* that compiles research results from investigations on the station. The latest edition collected 312 scientific publications between October 1, 2019, and October 1, 2020, “which brings publications to a total of 2,850 since 1999, with sources in peer-reviewed journals, conferences, and gray literature representing the work of more than 5000 scientists worldwide” (ISS Program Science Forum, 2020, 2).

Just like assessments with regards to costs, publications are not merely a matter of magnitude and must be situated in comparative contexts. The question about whether that number of publications is “too little,” “enough,” or “impressive” requires a substantive understanding of the actual process of conducing the experiments before engaging in comparisons. And, since there is no comparison outside standpoints, this takes us to some key characteristics of valuation.

## Valuation as Problematic and Indeterminate

Following Dewey, we do not appraise the unproblematic and the process of appraisal is continuous. The distinction between ends and means as temporal and relational is the key to his processual viewpoint: “Every condition that has to be brought into existence in order to serve as means is, in that connection, an object of desire and an end-in-view, while the end actually reached is a means to future ends as well as a test of valuations previously made” (1939, 43). In Ezorsky’s interpretation, she questions a naturalistic reading of Dewey, and restates that “the question ‘is it good?’ is continually reopened in inquiry, its significance reflecting the lack of finality and ongoing character of inquiry itself” (1958 ,19).

Studies of organizations have their own language to characterize what is going on with the ISS: hybrid organizations and evaluative uncertainty. Hybrid organizations are defined as “international, multi-sector phenomena with unclear sector accountability that often engenders unease and distrust” (Billis, 2010, 46). In terms of valuation, this literature also offers a tautological framing for the problem: “the more external audiences constitute the institutional environment of a set of organizations, the more plural is the organization’s evaluative landscape” (Brandtner, 2017, 218). In the managerial mode of these perspectives, hybridity, plurality, and uncertainty are problems to be overcome. On the contrary, pragmatist and hermeneutic approaches do not seem to have such concerns, which does not mean that they simply dwell on complexity. They enable different ways to approach it.

A key aspect to keep in mind when using these frameworks with the ISS is to interrogate comfortable generalizations. Critics and supporters see different things in the characteristics of the station outlined above. Again, this is a an ideal-typical exercise in the Weberian sense, which entails accentuating features encountered in actors and elevating them to the status of a category. I am not treating them empirically as fixed positions, and my aim is different from that of a normative philosophical approach that rejects or accepts arguments subjecting them to quality and rigor standards (Schwartz, 2020). My task is to see the social work these positions do for their constituencies. I am also aware that the order in which I place the statements might give the impression of giving “the last word” to one set of views, which is not the case.

Having said that, where critics see anxiety of pertinence in the multipurpose nature of the ISS, supporters see plenitude of purpose. This very distinction – sometimes imperceptible for those involved in the discussions – is of profound consequences for the valuation of all aspects of the space station. In other words, for the former, since there is no “actual” rationale to put people in space, the program had and has to “promise to do everything for everybody,” as stated by a program official, hence confirming the critics’ premise about the lack of a compelling rationale. Meanwhile, for supporters, the multipurposefulness of the ISS is nothing but the confirmation that spaceflight is important as it addresses vital areas of politics, economics, inspiration, technology, and science. Along these lines, both sides recruit the always malleable category of “the public” in support of their convictions.

Even a more specific constituency like “the scientific community” has been used to make claims for both sides, and – consequently – requires distinctions. In relation to the ISS, the scientific community includes skeptical external observers and a mixture of disappointed and hopeful users of the platform across fields.

The most vocal critics have been physicists in space science who claim that human spaceflight produces insignificant science (Matthews, 2012). In the early days of *Space Station Fredom,* James Van Allen published a detailed study (1986) countering NASA's case for a space station. Years later, he re-stated his position: “In a dispassionate comparison of the relative values of human and robotic spaceflight, the only surviving motivation for continuing human spaceflight is the ideology of adventure” (2004, 822). Nobel laureate Sheldon Lee Glashow made the following declaration about the arrival of the first particle physics detector to the ISS (the Alpha Magnetic Spectrometer): “It will finally be true that there will be real science on the space station, something that has never been true before — it’s been like high school experiments and a bunch of silliness” (Interviewed by Greenfieldboyce, 2011). More recently, during the 20th anniversary of the ISS in November of 2020, a BBC reportage cited British Royal Astronomer Lord Martin Rees, another noted long-standing critic of the space station:

“None of the hundreds of people circling around in the International Space Station have done any worthwhile science sufficient to justify even a tiny fraction of the money the Shuttle and space station have cost us (…) The space station makes the news when [astronaut] Chris Hadfield sings or when the toilet doesn't work. What actually makes the newspaper headlines are the marvelous pictures from the Hubble telescope and those of the surface of Mars and Jupiter and Titan, all obtained robotically” (Cited by Hollinghan, 2020).

In response to this kind of statements, medical researcher and former Chief Scientist at NASA’s Human Research Program, Mark Shelhamer defends human spaceflight and the role of science in it. While acknowledging that physical sciences “have far outpaced the life sciences in discoveries from space research,” he points to a presumption behind the criticisms: the belief that discoveries in the physical space sciences are more important than those in the space life sciences. For him, “it is not inherently obvious that, for example, knowing the composition of galaxies is more important than understanding how humans respond to altered gravity and other stressors of space flight” (2017, 38). He further specifies aspects of experimentation in the life sciences on the ISS:

“We have yet to send an astronaut into space, for an extended period of time, with the sole purpose of performing biological and physiological experiments, noting anomalies, and exploring new leads. This is how laboratory science is generally performed in this field, but we have yet to have the resources to do it this way in space. A reasonable case can be made that this research community should be congratulated for performing as well as it has, given these many complications” (Ibid).

This brings us to the communities that conduct research on the ISS, particularly microgravity experiments. Here, the mixture of disappointment and enthusiasm is a product of the troubled paths of the program and the difficulties in accommodating their research requirements.

During the *Space Station Freedom* process, the U.S. government appointed a Task Force on the Scientific Uses of the Space Station (1981-1987). This team, led by Stanford Professor Paul Banks, assessed the potential of the station as a versatile scientific laboratory requesting specific infrastructures, more saliently, a centrifuge that would have allowed biological studies on different gravity levels. Most of the ideas proposed by this committee were defunded during the redesign of what became the ISS. This descoping, to this day, represents a grievance for this community of researchers. In addition, as summarized by a program official during our interview in 2015, “promises were made too early, building took too much time, and then the interest faded.”

In 2010, as an effort to regain this interest, NASA commissioned the National Academy of Sciences to produce a decadal survey. *Recapturing a Future for Space Exploration: Life and Physical Sciences for a New Era *(National Research Council, 2011) is a document in which “the community” became an institutionalized voice which agreed on a set of scientific priorities defining areas, recommending a research portfolio and a timeline for conducting that research, and identifying facility and platform requirements. This is a small community of researchers that has struggled for a place in the research landscape of human spaceflight due to lack of funding and a peripheral role in the larger exploration goals for the agency during the years of assemblage. Since 2015 I have been participating in their meetings and town halls, where tribulations of the past about the agency’s neglect towards them reappear, and where they hope new possibilities can emerge again. As I write this in 2021, they are in the process of crafting their research agenda for the next decadal survey. They are now part of NASA’s Science Directorate and are being directed to frame their projects in terms of “campaigns” following the model of astronomy and planetary science (Alexander, 2017) as a way to overcome the scattering that currently prevails and to think of investigations for future Lunar exploration.

Beyond scientists, supportive arguments cover the “good for” and “good in itself” spectrum. Next to the general invocation of science, the so-called spinoffs are significant for this line of arguments. Two salient examples here are the technology used to purify water on the ISS and its applications in “global communities,” and the robotic technologies developed for space applied in neurosurgery (ISS Program Science Forum, 2020). And when pushed beyond the framework of usefulness, there are emanationist arguments about the inherent human drive to explore, the awe-inducing nature of space, its expansion of the human experience (summarized with different evaluative lenses by Schwartz, 2020; Elvis, 2021), and even claims about the experience of transcendence and self-transformation of looking at Earth from space (White, 1998).

A powerful player like Elon Musk, perhaps enabled by the affordance of wealth that seems beyond accountability, says: “Ultimately, because it is cool.” When I asked him during a Q&A at the ISS R&D Conference in 2015 how he would describe the scientific value of the ISS, he replied:

“I’ve really spent most of my time thinking about how to get to the space station. To be honest, I actually hadn’t even seen a proper movie about the ISS until I went to see a preview of the new IMAX film that is coming out, and it is amazing (…). It is the only laboratory that is above the Earth’s atmosphere, and you can learn a lot about basic human physiology and do experiments that you cannot really do in any other lab, and you can bring scientists up and they can work in this very unique lab. There is a lot to be gained there. And I think you can’t just ignore the coolness factor of it…it’s the coolest thing going on in space and there is a lot of value in that” (Transcription ISS R&D Conference, 2015).

Next to those involved, there are very few scholarly works that have addressed the valuation of the ISS. Among them is a special issue of the journal *Social Science Quarterly* in 2017 devoted to “Selling Space.” The agenda of the authors is to provide an evaluation of the ISS “beyond emotional appeals, vague generalities about the long-term value of space exploration, or assertions that the station contributes to peace between nations” (Bianco & Schmidt, 2017, 1151). Their aim is to show that the station can be evaluated “using yardsticks that are familiar to researchers across scientific disciplines, such as the likelihood that research projects yield refereed publications, the quality of publication venues, and generation of patentable findings” (Ibid).

However, their formulations rely on three problematic premises. First, and most consequentially, they treat the ISS as “canonical Big Science,” hence the recource to the familiar yardsticks for assessment. Second, based on those yardsticks, they provide a framing of deficit for the ISS: “citation and patent indicators paint an unpromising picture of station science” (Bianco et al., 2017, 1146), and “it will be years or even decades before these discoveries are refined into products or techniques that have a measurable impact on the lives of ordinary Americans—if indeed they ever do” (Bianco & Schmidt, 2017, 1152). And third, they naturalize the program’s concerns and consider that the main problem is one of “selling”: “how to persuade elected officials and the public to fund continued research, given that returns on this investment will take years or decades to materialize—if they ever do” (Bianco & Gaddie, 2017, 1139).

There are various intriguing gestures and claims in these papers. One paper begins with an epigraph by known critic Richard Feynman stating: “It bothered me a little bit that I never saw in any scientific journal any results of anything that had ever come out of the experiments on the [space] shuttle that were supposed to be so important” (Cited by Bianco & Schmidt, 2017, 1151). Regarding claims, the papers characterize ISS experiments, “like many other research programs,” as “lottery tickets” (Ibid, 1152). Specifically, they are “expensive normal science [in the Kuhnian sense] —combining high up-front costs with uncertain prospects of future benefits” (Ibid, 1158).

One analysis uses a multivariate model that “predicts the publications and patents from experiments as a function of time, project type, and affiliation of principal investigators” (Bianco & Schmidt, 2017, 1151-1152) and concludes:

“Two years after the start of data collection, the estimated likelihood that a scientific experiment yields an RP [refereed publication] is only about 0.25. However, at 100 months, this probability has increased to almost 0.75. This variation confirms that station science is indeed a work in progress. Relatively low publication rates reflect the simple fact that many experiments have not been operating long enough to generate publishable results—let alone complete the journal or patent review process” (Bianco & Schmidt, 2017, 1157).

Another paper proposes moving beyond publications, citations, and patents towards “waypoints that flag projects on the path to valued outcomes” (Bianco et al., 2017). The indicators here are also very outcome-oriented as waypoints, including follow-on funding, and are precisely treated as predictive of the “valued outcomes” of publications and patents (Bianco et al., 2017, 1146).

These papers reveal the recalcitrance of outcome as the focus, as well as some of the blind spots in assessments of the ISS that do not incorporate any understanding of the process and its particularities. Without data about “the how” of experiments, this is an inaccurate and homogenizing picture. Next to what I described above, time of operation in orbit is highly variable between experiments: some entail a single flight opportunity on which no publication of results is possible, others have sustained operations, and there are instruments that produce streams of data for years enabling multiple publications by large collaborations. Representatives of the program also erase all these distinctions when they state in various forums that the median time to publication in a scientific journal after a research activity is completed on the ISS is 1.5 years.

My claim here is not that a more thorough characterization can only be done qualitatively or ethnographically – which has been the core of my research – but that even the metric-oriented accountability of the program would require a different categorization of experiments and some information about the actual process of conducting them (Castaño and Bazán, in progress). Currently, the only metrics of this sort the program produces are about science utilization and crew time devoted to experimental activities in general. The publicly available databases for ISS research do not have any processual indicators, for instance, providing information about which experiments required sample return, specific involvement from crew members, which had hardware failures, needed repair, how many build on previous experiments, and which have outcomes that will live on in open data repositories.

 Sociologies of quantification understand the work that numbers do covering vast distances (Porter, 1996) in order to assess their limitations. In the words of Espeland and Stevens: “Measurement can help us see complicated things in ways that make it possible to intervene in them productively (consider measures of global warming); but measurement also can narrow our appraisal of value and relevance to what can be measured easily, at the expense of other ways of knowing (consider how education became years of schooling in American sociology)” (2008, 432). The most far-reaching of these limitations is that “the real easily becomes coextensive with what is measurable” (Ibid, 432). When the ISS program representatives talk about things that are “intangible” they are precisely referring to that which cannot be easily quantified, but also to that which they don’t even aim to represent.

Considering the characteristics of the ISS, the stakes and the conflicting interpretations involved, it is evident that there is no defectless framework to value ISS science. This problem is of course not exclusive to the ISS, and for that reason it can also shed light on key problems in complex institutions as I discuss in the conclusion. Aspects of ISS’ complexity enter or leave the valuation picture depending on the standpoint of participants and observers, and in some settings they do so with significant implications for the program.

## Valuation as an Interpretive and Perspectival Process

In the presentation of the *Journal of Valuation Studies*, the editors highlight that the plurality of regimes of worth is a pressing topic for social scientists (Helgesson & Muniesa, 2013). In principle, different implications can be derived from this plurality. For some, greater resilience (Lamont, 2012, 202) and innovation (Stark, 2009, 15) can be brought on by divergence of evaluative principles and orders of worth. Disagreement about what’s valuable, in Stark’s view, can make for “new value propositions.” He proposes to study the productivity of frictions and misunderstandings, but ultimately has organizations with a single type of outcome in mind which differ from a space station and other multi-jurisdictional institutions.

Despite the multiple nuances in his perspective, Dewey also did not have multidimensionality of outcomes in mind as the foci of valuation. In one of his examples, real estate “is appraised for the purpose of levying taxes or fixing a selling price;” medicinal treatments “to the end of effecting recovery of health;” and materials and techniques “to the building of bridges, radios, motorcars, etc.” (1939, 23). A plurality of regimes of worth also invokes a more fundamental problem: valuation requires categorization or typification. And, for less optimistic perspectives, objects that fall within categories “are shown to have often been more difficult to assess and therefore to have been given lower value and conferred lower status” (Lamont, 2012, 204).

Therefore, there are more specific tasks at hand when facing this plurality, particularly when the object is one that cannot ultimately fit in a single category. In the pragmatist move, the operations through which a quality is assigned to an object, in Heinich's terms, depend on the nature of the object being evaluated, on the nature of the evaluating subjects, and on the nature of the context of valuations. In the taxonomic inclination of her programmatic claims, there are categories of objects that undergo valuation: things, people, actions, and states of the world (2020a, 219). The ISS is a good case to think through the limitations of these compartmentalizations, considering not only the diversity of actors that make assessments, but what they mean specifically when they say “ISS” in general and “ISS science” in particular.

Pragmatist definitions of valuation then need to be complemented by the hermeneutic gaze towards modes of understanding and the ways in which actors and institutions carve out aspects worthy of their valuative operations, the interpretations involved in compartmentalizing or combining elements, as well as the temporal framing of the assessment. Dilthey beautifully phrases the different predispositions at stake here when he makes the following distinction: “How impatiently do we listen to many arguments, merely extracting the point that happens to be important to us practically, without any interest in the inner life of the speaker; at other times we passionately attempt to seize the innermost reality of a speaker through his every facial expression, his every word” (1900, 237).

However, the alternative to the managerialist language of organizational theory about hybrid institutions and evaluative uncertainty is not surrendering to a chaotic perspectivism where “everything is relative” depending on “people’s views.” On the contrary, the outlook informed by pragmatism and hermeneutics is one of systematic inquiry about valuation processes.

In the valuation settings for the ISS that I have examined in my work (Congress, the National Academies of Science, and media reports), I characterize statements in terms of what a particular group of people consider worthy as they select and highlight aspects of the broad ISS program and of science in particular. Who makes assessments about the scientific value of the ISS and its experiments? Voicing what kind of institutionally sanctioned spokespersonship? What evidence is considered and what are the domains under scrutiny? What are the underlying general understandings of science and of the experiments in these assessments? How are the impacts of ISS research characterized for ordinary people on Earth, for space exploration, and for specific scientific communities? What is their temporal framework in the assessment? What parameters are involved when they discuss data provided by the program?

Assessments of costs and number of publications are salient here since different claims can be made based on facts about the ISS. The program represents 14% of NASA’s total $22 billion budget, which in turn is approximately 0.5% of the total United States federal budget. Compared to the three examples cited in the introduction, the total reported cost of LIGO over 40 years was $1.1 billion; the total cost of finding the Higgs boson was around $13.25 billion (including the costs of building the Large Hadron Collider which includes other experiments and took a decade to construct), and the Event Horizon Telescope’s costs were between $50 and $60 million (Knapp, 2012; National Science Foundation Fact Sheet, 2016; Moon, 2019).

A statement like “a single year of ISS operations could pay for three LIGOs” is a clear stance regarding ideas of worth about the program and hierarchies between fields of research. Evidently, this is not how the actual administration of budgetary relocations works, but the maneuver of saying “this could have paid for this other thing” is always an indicator of positions, in this case, regarding governmental spending or investment (the choice of words here is also part of the discussion).

On the other hand, statements like “they manage to do so much with so little” would require considering that only 10% of the total ISS budget is devoted to experiments, that astronauts conduct just 40 weekly hours of science-related activities in the midst of multiple and demanding operational tasks, and there is only one uncrewed vehicle that can return samples to Earth approximately every six months.

The same holds true for the number of publications reported by the program. Without context and attention to the radically different nature of the endeavor, the 2,850 total publications resulting from the ISS seem unimpressive if compared to other programs. For instance, CERN publishes approximately 1,000 papers per year, and the Hubble Space Telescope reached its 10,000^th^ publication about a decade ago.

In terms of valuation settings where these ideas are mobilized with great consequences, the most important one is of course the U.S. Congress. The space program needs political justification and budgetary allocations, and in Congressional reviews everything is frequently sent back to the drawing board of its broadest justificatory frameworks. A 2017 hearing in the House of Representatives’ Committee on Science, Space and Technology provides a clear example of this dynamic. In the customary adversarial form of the hearing, representatives stand as implacable judges acting as custodians of the taxpayers’ dollars. On the other side are the representatives of the ISS and key stakeholders who are there to make their case providing written and verbal testimonies and being treated as witnesses. All of this while both sides converge in their underlying agreement on the importance of the U.S. space program.

William Gerstenmaier, the Associate Administrator for Human Exploration and Operations at NASA at the time, fulfilled his duty as spokesperson for the ISS. His testimony aimed to cover the range of disciplines and applications on the station that “ultimately benefits people on Earth.” As in many other instances when space station science is presented, the inventory is extensive: microbial systems, fluid physics, combustion science, materials processing, environmental control and fire safety technologies, education, Earth observation, clinical medicine, telemedicine, human physiology, cardiovascular research, bone and muscle health, neurovestibular effects, diagnostic instruments such as advanced ultrasound, exercise and pharmacological countermeasures, food and nutrition, immunology and infection, exercise systems, human behavior and performance, and visual impairment due to intracranial pressure. He also mentioned the “Station activities that can benefit humanity” in robotic microsurgery techniques, water purification technologies for developing regions, pharmaceutical studies and station-generated images that assist with disaster relief and farming. In medicine, he added, “the growing senior population may benefit from experiments in the areas of bone and muscle health, immunology, vestibular response and balance, and from the development of telemedicine techniques used to monitor and treat ISS crews. These telemedicine capabilities can be used on Earth to improve medical care to patients without requiring travel to a hospital or doctor” (Gerstenmaier, 2017, 2).

I transcribe these words at length to stress a characteristic about this program: the almost overwhelming list of research fields, activities and aims that it covers. In a setting defined by the pressures to quantify and convey short and timed answers to complex questions, where there is just no time for detail, this can sound like a ramble. Answering a question, Gerstenmaier, stated: “We all would like to have that ‘killer app’, or the ‘killer result’, but part of the problem is the complexity and the variety of research on space station.”

He was followed by Mary Lynne Dittmar, Executive Director of the Coalition for Deep Space Exploration at the time, and Eric Stallmer, president of the Commercial Spaceflight Federation. Their role was to make the case for public-private partnernships on the ISS and to portray a future where the continuation of the platform is left to private companies. Finally, to defend the scientists’ point of view, Robert Ferl, Distinguished Professor and Director of the Interdisciplinary Center for Biotechnology Research at the University of Florida intervened. Ferl has 25 years of experience in spaceflight research, played a central role in the 2011 Decadal Survey at the NAS, and co-chairs the one currently underway. He described his recent experience with plant biology experiments on the station:

“I wish to share with you a very positive view regarding the current status and activities on the ISS. A view enriched by experiments conducted over the last few weeks on the space station, monitored in real time from KSC [Kennedy Space Center] three weeks ago with [astronaut] Peggy Whitson doing our stuff on orbit. We spent last week at Glenn Research Center downloading in real time information from the life microscopy module on orbit, and our samples landed in the Pacific this weekend. I can tell you there is essentially no better time to be a spaceflight researcher than right now given the capabilities of the ISS. The demonstration and the evolution of the quality of experiments as they move from the early days and the space Shuttle era into the space station era is dramatic…” (Transcription recording U.S. House of Representatives Committee on Science, Space and Technology, 2017)

In the round of questions that followed the testimonies, representative Dana Rohrabacher, who claimed his place in history as the vote that saved the space station program back in the nineties, interrogated Gerstenmaier about the cost of assembling the ISS for the United States and the annual operation costs of the station for NASA, and used his five minutes to make a statement:

“I am not sure if the scientific research that has been done on the ISS has justified the expense of the 67 billion dollars that we are talking about. I am not sure that is the case. But there are other factors rather than just scientific research that come into play, and Space Station certainly renewed our national self-assuredness that we could do great things. It’s hard to put a price tag on that. When people are demoralized, they think they can’t do great things, they don’t do great things, and that costs certain amount of money. Let me also note that the space station played and continues to play an important role in creating an image of peaceful cooperation between the two countries that were willing to destroy each other and to destroy the planet for the 40 years prior to building the space station […] The promises, there were many promises, that cancer would be cured. I sat here and listened to them. Well, I never did buy that. I hoped it would be true, but I never really counted on that, but I hope that we can say that when we write our history of what has been accomplished in the last century, Space Station will be up there on the list of great accomplishment and of things that show that there is a better way for mankind” (Ibid, oral statement)

The hearing encapsulates the diversity of hopes and categorical claims on both sides of valuations about the ISS, as well as the underlying agreements that sustain the undertaking. As compatriots of the cause of extending the life of the ISS program, the four witnesses unanimously stood there to defend what Dittmar summarized with these words: “We need more time to allow for these things to develop.”

Statements about the scientific worth of the station are made here for and on behalf of citizens, taxpayers, scientists, the commercial sector, and the ever-malleable public. In various moments, this public is a diligent accountant, an inspired spectator, a skeptical critic, or is represented in the “human us” that goes to space. These are all clearly rhetorical maneuvers in the sense of “speech designed to persuade” (Burke, 1950), but this acknowledgment cannot slip to a mere strategic reading of the situation, or to simplistic assumptions about the actors as only guided by self-evident interests. Decoupling the notion of value with that of interest is indeed part of the pragmatist agenda, but it takes hermeneutic work to engage in nuanced empirical inquiry. A central part of this inquiry entails looking at how languages of valuation become institutionalized.

## The Institutionalization of Valuation Frameworks

The characterization of the ISS so far shows that there is no fixed standpoint from which to assess this large complex program of multiple moving pieces and with so many activities and valuations attached to it. The problematic, indeterminate, perspectival, and interpretive nature of valuation leads to an inquiry about how some modes of assessment sediment more successfully than others over time, that is, to a question about institutions. Here, as sociological updates on hermeneutics and pragmatism respectively, the works of Glaeser and Bargheer revive these perspectives in pertinent relation to this topic.

Glaeser’s work on the secret police and the end of the German Democratic Republic (2011) culminates in what he calls a Hermeneutic Institutionalism (2014), expanding the imaginaries of Vico and Herder. Glaeser proposes a theory of understandings and the ways in which, via recurrent validations, they come to be objectified as transposable forms, hence forming institutions. There is an empirical observation and an agenda posed by this work that I take up in my approach to the ISS. In good hermeneutic fashion, and resulting from detailed ethnographic work, Glaeser notes that “both individuals and collectivities of people typically operate simultaneously with a variety of understandings which may indeed be locally integrated with each other, but which must also be allowed to stand in ambiguous, ambivalent and contradictory relationships to each other” (2014, 218). Two linked research puzzles emerge from this observation: “a reconstructed social hermeneutics will have to turn assumptions about unity or consistency of understandings into researchable problems,” and “theories emphasizing the plurality of competing understandings need a way to account for selection” (Ibid, 236).

In the case of pragmatism, Bargheer’s work on the histories of bird conservation in Britain and Germany aims to reformulate Dewey’s theory of valuation in terms of institutions. Dewey himself stressed the need for this line of inquiry: “When current theories are examined which, quite properly, relate valuation with desires and interests, nothing is more striking than their neglect so extensive as to be systematic of the role of cultural conditions and institutions in the shaping of desires and ends and thereby of valuations” (1939, 64). Bargheer offers a more traditional grasp on institutions as “arrangements of rules and resources that organize action” (2018, 26), but his crucial insight for the study of valuation relies on the detailed empirical work needed to demonstrate how the value of something results from its relational position within a set of practices and institutions (Ibid, 5). In addition, following interpretations of the relationship between means and ends in Dewey (1939) and Joas (2000), Bargheer shows how ends are not the only carriers of value while means have a merely instrumental function (2018, 21). Ends, above all, are not a state but a process: “As long as they are unachieved, it is not the end result of action itself that motivates agency but the prediction or anticipation of this end” (Ibid, 22).

I take from both works the tasks they pose and as they move beyond the simplistic conceptualizations of institutions in terms of “incentive structures” or “rules of the game.” As described above, the purposeful multi-functionality of the ISS is at the core of this institution-forming process, but – in the midst of this seemingly untamable complexity and diversity – there is an unstated and underlying convergence: the prevalence of outcome-centered logics of assessment. Next to notions like “benefits for humanity,” “impacts,” and “breakthrough,” the idea of “return on investment” has taken over analyses to make claims about the ISS.

Congressional overviews provide a powerful setting for the recurrent validation of this framework to such an extent that it has almost become the only way to talk about the value of the station. Some ends, returning to Dewey, become “so standardized by custom that they are taken for granted without examination, so that the only problems arising concern the best means for attaining them” (1939, 43). As an official told me during an interview in 2015: “This is just the way we have come to think about the program.”

The condition of possibility for this framework leads to broader questions about how these ideas came to pervade assessments of governmental spending, something that is a line of inquiry on its own. For now, here I point to the fact that economists certainly have “the ear of the prince” (Abbott, 1999, 194), and bureaucracies are largely staffed with economists or graduates from business schools where these ideas also become transposable forms and develop into “just the way things work.”

These understandings, that also have “no time to waste on detail,” require a unidimensional grasp on the objects under assessment which, in turn, enables commensuration among otherwise disparate aspects and draws a line between what is relevant and what is not. Again, in the language of organizations theory, “not all the conceivable ways for an organization to be considered successful by its audiences—such as a corporation being judged the most family- friendly place to work or as having the most faithful employees—are necessarily operationalized in some consequential evaluative measure” (Brandtner, 2017, 217). The solution, from this logic, is procedurally clear: just establish benchmarks and generate evaluative certainty. Here, the need for a notion like “return on investment” is beyond question because it provides that institutionally sanctioned certainty.

There are two consequences for the ISS in the institutionalization of these understandings. The first is that it consolidates an image of the program in terms of shortfall. One can simply attribute this to the nature of the political process, but the need to constantly justify the station and to defend its funding becomes all that officials see about the ISS and their own work. The difficulties in “putting a dollar tag” on the multiple activities ends up meaning that most of what these officials do on an everyday basis does not have value even before their own eyes.

The second consequence is that the logic of “return on investment” facilitates a larger political process shaping human spaceflight and space generally labeled as “commercialization.” The process is complex and various specialists have discussed it from several angles (Macauley & Toman, 1986; Klinger, 2017; Teong, 2017). In one key dimension, space commercialization appears as an inexorable path and is a textbook case for what Mazzucato (2018) discusses as “public-private sector myths”: under a naturalized idea that governmental programs are large and inefficient in light of a set of assumptions, private actors are invited to save the day. Weinzierl provides a good summary of these assumptions: “The vulnerabilities of centralized control will be familiar to any economist: weak incentives for the efficient allocation of resources, poor aggregation of dispersed information, and resistance to innovation due to reduced competition” (2018, 176). Under this framework – and even with the rawness of considering that NASA was created as “a response to classic market failures” (Ibid, 179) – the agency is portrayed as inefficient in achieving its own goals. While not entirely convinced of the prospects of the space economy, Weinzierl sees a trajectory and cites Launius when he makes a historical analogy of space exploration and the commercial aviation industry where “the US government played a critical role in basic research in the mid 20th century while leaving the operation of the aviation sector in private hands” (Ibid, 183). As it is clear in other contexts, political decisions that could be subject to deliberation, become “the unfolding of history” or “mere technicalities” under these modes of understanding.

In sum, “return on investment” as a transposable form, in Glaeser’s terms, is institutionalized as it becomes the hegemonic background of assessments and restructures the relationship between means and ends for the ISS.

## Valuation and Time

Historians have shown how the temporal mode of space is the future (Siddiqi, 2011; McCray, 2012; Geppert, 2018). However, this idea requires further specification in light of the puzzles of valuation. Even if the various actors diverge in their commercial, political, and scientific ideas about the content of what is valuable, they all coincide in their future-oriented gaze.

The institutionalized logics of valuation for the ISS are not only modes of assessment based on what was and has been. They are, above all, statements about what could be, and ought to be. From the start, for its supporters, the station exists in constant anticipation towards a better future: one of space exploration, knowledge and technology that benefits life on Earth, and more generally regarding science, “the breakthrough that shall be.” And here, using Abbott’s words, is where “the future encodes itself into the present” (2019). In this section I take broad inspiration from his work about time in sociology (2001, 2016) and his current thinking about the fact and value problem as embedded in the flow of social time (2019).

In valuation processes what ought to be is outside the present. While pointing towards it, pragmatist and hermeneutic approaches to valuation have not fully grasped the puzzles of temporality in this process. Dewey sets the stakes of the matter but does not unravel them when he states that valuations “occur only when it is necessary to bring something into existence which is lacking, or to conserve in existence something which is menaced by outside conditions, valuation involves desiring. The latter is to be distinguished from mere wishing in the sense in which wishes occur in the absence of effort” (1939, 15). Here, once more, a classical strand of the hermeneutic inclination can take us a bit further. Again starting with Dilthey (1862), the process of understanding is always historical in nature, and interpreting something (or someone) entails a commute – in the flow of time – between their temporalities and those of the interpreter.

The economization of metrics and the institutionalization of the “return on investment” framework again play a significant role here, but they are not the full picture. As Muniesa and Doganova have shown, the language of future value is inherent to the financial view on things. While this is an insightful opening of new conversations about time in studies of valuation, the authors are critical of sociologists reinforcing “financial reasoning” by treating the forward-looking concept of value uncritically (2020, 109).

Taking distance from this view, the future invoked by ISS is not only a political technology that manipulates uncertainty for present gains like in their acute description of the world of finance. It would be fairly easy to analyze things only in those terms when it comes to space station science, but there are also moral convictions and shared emotions about the past and about possibilities not yet realized (Quéré, 2015, 176). In the coordinated work of thousands of people in dozens of different ground locations that sustains the ISS, meanings about the overall endeavor come in many forms: people just doing their job, aiming to make a profit, putting up with the limitations to conduct their experiment in an extreme environment for which they do not have any particular fondness, fitting some scientific activity in their passion for space, or living their childhood dream and working for something they perceive as transcendent beyond the finitude of their lives. Of course, some of these meanings live in the same individuals who do not experience their coexistence as contradictory.

Additionally, writing about ISS science “in the midst of it all” – that is, as things are unfolding – means that uncertainty is an actual feature of the endeavor. In the terms of the “Selling Space” analysts, “it is too soon to say whether the science on the ISS will yield transformational breakthroughs, solid science—or nothing at all beyond journal articles and patents” (Bianco & Gaddie, 2017, 1141). In a more optimistic prognosis, “while we cannot speak to the prospects of any particular project, many experiments are clearly producing noteworthy results. The rate of such outputs should improve significantly over time” (Bianco & Schmidt, 2017, 1158).

As confirmed by multiple interventions of ISS officials in various settings, they live with a hope: beyond the publications and applications of the thousands of experiments, something along the life of the ISS has got to be a “big breakthrough,” or that “killer app” will show up somewhere. This forward-looking perspective is omnipresent when it comes to the station both in appeals to emotion and in the language of forecasts: “it is forecasted that ISS stakeholders will see returns never before realized when commercial crew capabilities allow an additional U.S. crewmember in 2017. The additional ISS lifetime beyond 2020 will allow greater amounts of research, new discoveries, and benefits, especially in areas of ‘next-generation’ research that has already been conducted on ISS over the last decade” (Ruttley et al., 2017, 1172-1173).

In a nutshell, what ought to be with the ISS is that which is yet to become. The distinction between the supporters and the critics now can be placed along the lines of those willing to wait (with varying degrees of hope), and those for whom it is already too late (because they never had patience in the first place).

Assuming for a moment that the substantial and the unimportant can only be distinguished from each other retrospectively – something that historians would dispute because the past is never fixed – it is worth considering conjectural scenarios about what will be on the record according to whom when it comes to the ISS. While speculative, some questions are pertinent here as 2024 is approaching and there are several scenarios for the end of life of the station: commercial companies taking over management of the program (and “freeing” NASA’s budget to return humans to the Moon and send them to Mars), splitting elements that can remain in orbit, or fully deorbiting and destructing the platform (NASA Office of the Inspector General, 2019a, b).

What part of the process of conducting experiments in multiple fields on the ISS will be left out and will be assigned a particular type of relevance and by whom in these scenarios? What if a “ta-da!” moment were never to occur? Where are the sources for future historians to interrogate triumphalist or defeatist accounts of the ISS? If an undoubtedly significant breakthrough were to occur, will there be a retrospective attribution of coherence and linearity, and would there be no need to deal with the processual complexities of the station beyond some anecdotes? If that is not the case, how would accounts of the intricacies of the ISS enter the picture? This brings us back to a foundational gesture in the early days of sociology of science: countering the notion that when “things go well” (when science is “successful”) there is no need for a sociological account (Bloor, 1976). Of course, the possibility of doing sociology of science entails overcoming this notion, as well as empirically examining how distinctions between “successful” and “unsuccessful” science are made.

If what ought to be on the ISS is what is yet to become, then the present is devalued. Namely, the ongoing everyday tasks, the daily achievement of operations becomes the almost invisible background against which stands what is declared important. Here, there is no room for the artisanship of decisions that enable better watering of plants aboard the station; the drama of analyzing ambient blood samples in Houston hours after they landed in a Soyuz capsule in Karaganda; the dedication of contractors checking the temperature of frozen samples as they are being returned to laboratories; the loss of equipment and years of work in a cargo rocket explosion; and the lifetime work of thousands of scientists in their various fields. Amidst the ruling outcome and metric-centered assessments, my work aims to archive the process and that in itself is a stance. Whether it will be interrogated in the future and recruited for an “inspiring story,” or used as an unnecessary romanticization of an “expensive turkey in the sky” is unavoidably defined by time, perspective, and standpoint, and is beyond my control. Still, using Dewey’s words again as he talked about the non-human sciences, “nothing happens which is final in the sense that it is not part of an ongoing stream of events” (Dewey, 1939, 43).

## Conclusion

In the epilogue of his memoir, former astronaut Scott Kelly wrote:

“I think sometimes people want to hear there was one profound scientific discovery from the 340 days I spent circling the planet - something that struck me or the scientists on the ground like a cosmic ray through the skull at some climactic moment during my mission. I don’t have anything like that to offer. The mission that I prepared for was, for the most part, the mission I flew. The data is still being analyzed, but the scientists are excited about what they are seeing so far” (2017, 416).

The “ta-da!” expectation is omnipresent for the ISS given the hyperbolic nature of its promise, its costs, prevailing ideas about scientific discovery, and its inadequate grouping with institutions dedicated exclusively to scientific research. However, a different picture emerges once we examine the complexity of activities, the contrasting predispositions of the assessments, and the hegemony of instrumentality in the most consequential amongst those assessments.

The problems posed by the ISS make it sharply clear that to study scientific research sociologically, one also needs a theoretical approach to institutions and valuations. The exhaustive ethnographic gaze at scientific practice, following its workings, meaning-making practices in the everyday work of experiments cannot be disentangled from an equally detailed account of the actors and settings that, with powerful implications, make claims of worth about science. Institutions and valuations here are not the background doing the explanatory work for science. They are objects of inquiry in themselves because that is where – to use a much-loved expression for some in the sociological study of scientific practice – science as a process is actually “black-boxed.”

Stepping outside the sociology of science, this argument can be extended to the sociological study of other multi-jurisdictional institutions. Building on this approach to the ISS, I am working towards a more general conceptual framework for what I call *iridescent institutions*. The concept of iridescence refers both to the physical structure of an object that causes light waves to combine producing a prismatic display of color, and to the visual effects of that light interaction depending on the viewing angle. For the ISS, the concept combines the station’s intrinsic complexity and its various frameworks of valuation. In broader terms, an iridescent institution has three qualities. First, it is unlikely and accommodates multiplicity in dynamic and intricate arrangements. Second, it appears as different things when seen in different contexts and times. Third, as a complex set of possible things, it is elusive, its moral claim to necessity is in the future while working with the constraints of the past and the present.

An iridescent institution exists in the realm of possibility and can never fully deliver, and the question of when and how it has fulfilled its ambitions is never fully settled. Yet it works in its many-colored forms. And working – in the quotidian details of life – is its ongoing achievement. In consequence, an iridescent institution must be explained by integrating an account of its procedural heterogeneities and the changes in its viewing angles. This approach offers an analytical scaffolding for the study of institutions that fulfill varied principled tasks under constraining conditions and are subject to the evaluative gaze of multiple stakeholders.

In this context, taking the issue of temporality seriously in valuation processes is another pending analytical agenda that entails examinations of how valuation frameworks institutionalize approaches to time, as well as what time and perspective do in our own analysis regarding the moment where we meet our objects of inquiry as sociologists.

Regarding our stance in the midst of this iridescence, and going back once more to Heinich’s programmatic claims for a sociology of valuation, she states that “it has to remain free of any kind of normative perspective” regarding actors’ conceptions and opinions. In her words, “the sociology of valuation has nothing to do with helping actors in resolving conflicts, providing clues for good judgements, or fostering democratic ways of making decisions. The purpose of the sociology of valuation that we advocate here should remain a purely epistemic one, aiming not at making valuations acceptable but at discovering and analyzing how actors decide whether a valuation is acceptable or not” (2020b, 79). There is a Durkheimian default setting here to which many sociologists would not own up, and that - when militantly deployed - can have erosive consequences for the endeavour of doing sociology.^1^ Issues of objectivity are as old as our discipline and the scientistic anxieties on which it was founded, but the reaction to ideals of neutrality cannot simply be a politicized stance.

There are varying degrees of comfort that social scientists can afford as they observe the passage of world views before their eyes, without feeling morally concerned or existentially threatened by them. Different from issues of inequality of violence (which cannot be studied without condemnation as the starting point of ellucidation), space stations and space science could seem like milder topics. However, what I have illustrated above is that the contentiousness of valuation about the ISS and human spaceflight more broadly entails profoundly political and moral problems that are not at all weightless. Very serious issues are at stake and range from spiritual convictions about our place in the universe as humans, the short- and long-term implications of governments entrusting “the keys to the cosmos” (Dovey, 2019) to private companies, the allocation of public money in light of competing needs, the environmental impacts of venturing beyond this planet, to the accountability needed for publicly funded science.

The rigorous sociological task here contemplates a detailed account of the objects of assessment in as many of their dimensions as possible, which also implies taking the various positions seriously going from a concrete setting like a Congressional hearing to more general heuristics like the one provided by the supporters and critics distinction. This is a way to capture the iridescence and to analyze its stakes and implications. Constantly asking how each side of a contention would use a given observation to support their conviction is an exercise in analytical discipline. Having said that, none of these tasks happen from an epistemic and moral Himalaya and here are unavoidably normative positions in the selection of processual grounds of valuation and the problematization the “return on investment” logic.

Most of what matters is lost when we do not consider processes, and when we simply allow for the categorization of that which cannot be quantified as “intangible.” In the case of the ISS, this leads to consequential misrepresentations. If budgetary allocations always depend on expectations of “a big bang for the buck,” that ends up devaluing the research currently being conducted on the station as well as the daily operations in ground and orbit maintaining the platform.

The acceptance of “return on investment” as the ruling ground of valuation also comes at a cost. When ends are given in advance, there is little room to reopen deliberations. The line between luxury and need when it comes to human space exploration is a profoundly political and moral question that cannot be solved merely via cost-benefit analysis. And here the notion of human spaceflight as a universally inspiring endeavor, which is part of the convictions of some supporters, cannot be taken for granted to do the job.

In a striking resemblance with the divisions during the Apollo program era regarding the Vietnam war and the civil rights movement (Maher, 2017), on May 30, 2020 “the dawn of the commercial era of human spaceflight” (Drake, 2020) took place in the midst of unprecedented adversity and social upheaval in the United States. The launch of two astronauts on a SpaceX capsule from Cape Canaveral for the first time in almost a decade took place during the global Black Lives Matter protests against police brutality and in the midst of a devastating global pandemic. Both events seemed to be happening in different worlds, and if anyone had inquired with the protesters about the launch, they would have been more vocal critics than the physicists or astronomers cited above.

In our moment, using Van Allen’s word, human spaceflight runs the risk of indeed becoming the “adventure sport” of a few. In this case, of powerful company owners that are drawing futures in space for those who can afford them (Tutton, 2020), have their joyride, make a profit, get credit for being the ones advancing spaceflight, and do so using government infrastructures, contracts (NASA Office of the Inspector General, 2019a, b) and expertise (Berger, 2020). As these interests come to predominate and offer the mirage of “the business case for space” and “opening space for everyone,” there is a need for wide-ranging conversations about the meanings of human spaceflight and the place of science within it.

My study of the ISS for the past six years has involved conversations with almost two hundred actors involved with the program. My aim has been to make those conversations mutually meaningful reopening the levels of analysis about the station with a hope for broader considerations and more weighty deliberations. My task is not to give the program the metrics they need to make their case before Congress; to convince the skeptics of the value of human spaceflight or to confirm their ideas that “at the end of the day” it is all just about money and power; or to add a piece of sociological evidence to the dearly held convictions of the supporters. In other words, I do not intend to provide “the right content” for these valuations. Dewey knew that no abstract theory of valuation can be put next to existing valuations as “the standard for judging them” (1939, 60). However, as a process that will never be perfect, valuation can be less imperfect by bringing elements into systematic relation with each other, and by questioning what seems impermeable to debate. These are certainly grounds for a fruitful and continued confluence between pragmatism and hermeneutics in sociologies of valuation.

## Footnotes

1 Thanks to a comment from a reviewer, I learned about a significant development that took place between the time I finished writing this paper and this publication. In February 2021, Heinich, along with other scholars in France, created the *Observatoire du décolonialisme et des idéologies identitaires* (Onishi, 2021), and in May 2021, she published the leaflet *Ce que le militantisme fait à la recherche*. Both products cross several lines disqualifying social scientific works in France about forms of inequality related to colonialism, gender and race as some kind of “American woke fashion,” as “contaminating” French academia, and as “discovering the obvious.” A serious engagement with these issues and with Heinich’s dense package of misconceptions would require a research paper of its own, and some French scholars have already articulated criticisms to which I subscribe, particularly regarding her own militancy and her misreading of the Weberian “axiological neutrality” argument (Baubérot, 2021; Saint-Martin and Hardy, 2021). But I felt compelled to add this footnote as it shows the dangers of the Durkheimian default setting when it entails complete blindness to standpoints of privilege that treat many people’s oppression and struggles as mere landscape to be described, and serves as a sobering reminder that there is nothing “purely epistemic” in self-proclaimed stances of neutrality in valuation disputes.
